# Freiburg Neuropathology Case Conference: Back Pain and Rapidly Progressing Gait Disorder in a 70-year-old Patient

**DOI:** 10.1007/s00062-020-00945-8

**Published:** 2020-08-26

**Authors:** D. Erny, U. Taschner, J. Nakagawa, H. Urbach, M. Prinz, C. A. Taschner

**Affiliations:** 1 Department of Neuropathology, Medical Centre, University of Freiburg, Freiburg, Germany; 2 Department of Neuroradiology, Medical Centre, University of Freiburg, Breisacher Straße 64, 79106 Freiburg, Germany; 3 Department of Neurosurgery, Medical Centre, University of Freiburg, Freiburg, Germany

**Keywords:** Spinal metastasis, Chronic recurrent multifocal osteomyelitis, Spinal plasmocytoma, Spinal tuberculosis, Spinal lymphoma

## Case Report

A 70-year-old patient presented with back pain over the last 3 weeks that was initially controlled under analgesic medication. A weakness of both legs and exacerbation of the back pain resulted in hospitalization. On admission at a district hospital, the patient was still able to walk with the help of a walking frame. There was no known medical history of malignant diseases. Several years earlier the patient had suffered multiple left-sided embolic infarctions that resulted in a transient amaurosis and a discrete weakness of the right limb. Over the past few months, the patient had experienced an unintentional weight loss of 10 kg.

**Fig. 1:**
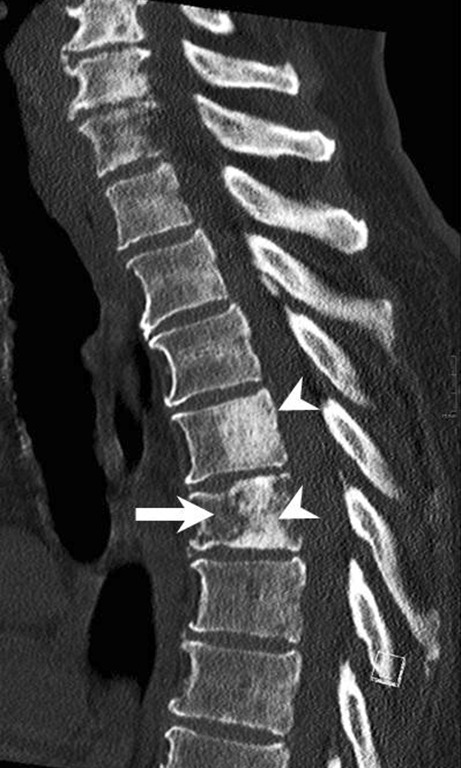
Sagittal reconstructions of computed tomography images of the thoracic spine in bone window setting showed increased density of large portions of the vertebral bodies of Th4 and Th5 (*arrowheads*). The vertebral body of Th5 showed a lytic area in its ventral portion and a compression fracture (*arrow*)

**Fig. 2:**
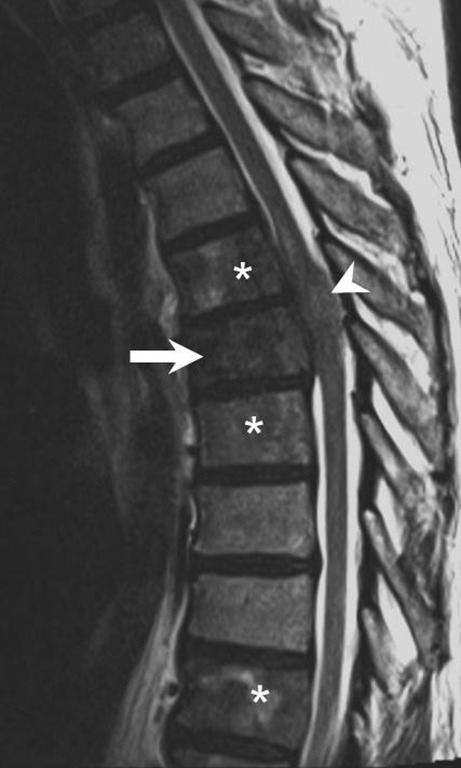
Sagittal T2-weighted images show patchy heterogeneous signal intensities in the vertebral body of Th5 (*arrow*) as well as within various other vertebral bodies of the thoracic spine (*asterisks*). An intraspinal, perimedullary lesion was found that showed isointense signal when compared to the myelon (*arrowhead*)

**Fig. 3:**
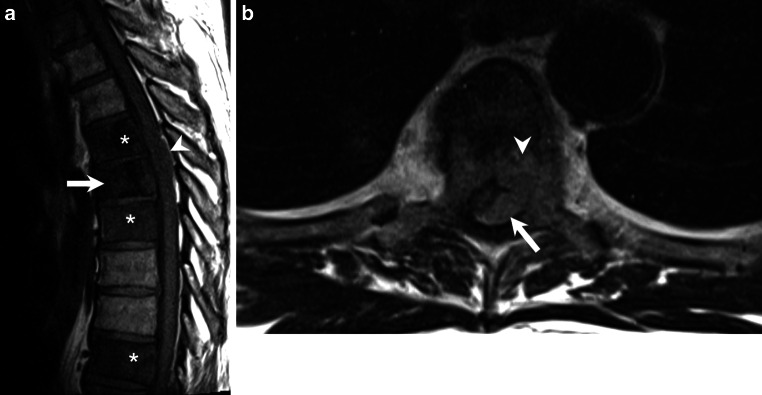
Non-enhanced sagittal T1-weighted images (**a**) showed decreased signal intensities in the vertebral bodies of Th5 (*arrow*), as well as Th4, Th6, and Th9 (*asterisks*). The intraspinal, perimedullary lesion clearly visible on T2-weighted imaging was hardly perceptible on sagittal native T1-weighted images (*arrowhead*). On non-enhanced axial T1-weighted images (**b**) at the level of Th5 the lesion appeared slightly hyperintense and seemed to be located in the epidural space (*arrow*) and showed infiltration of the vertebral body (*arrowhead*)

**Fig. 4:**
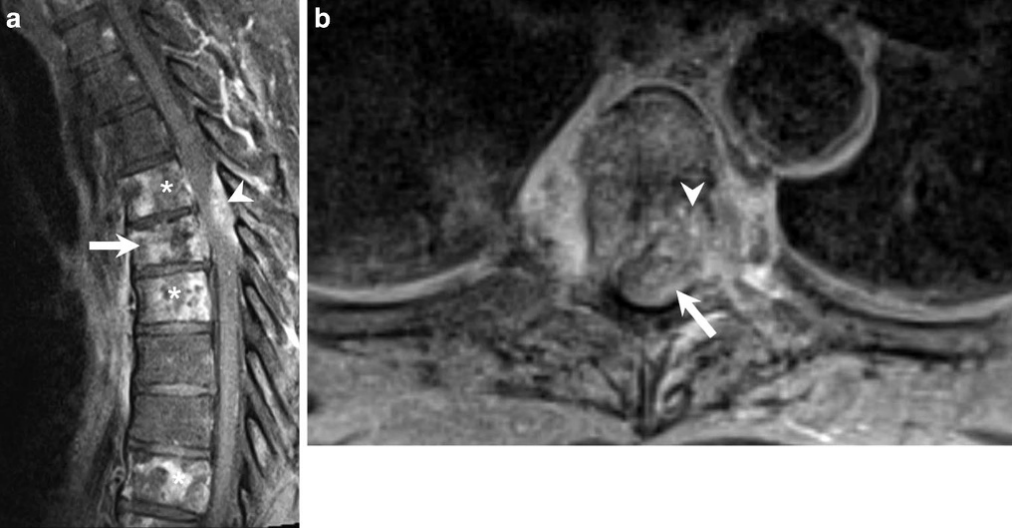
Sagittal T1-weighted images after administration of gadolinium (**a**) showed a marked yet patchy pattern of enhancement of the affected vertebral bodies (*arrow* and *asterisks*). The epidural lesion exhibited homogeneous contrast enhancement and displaced the myelon ventrally (*arrowhead*). On axial T1-weighted postcontrast images (**b**) the epidural portion of the lesion compressed the myelon (*arrow*), and the infiltration of the vertebral body (*arrowhead*) could be appreciated

After 3 days of hospitalization the patient suffered an acute deterioration of the neurological status and was transferred to our neurocenter. He now presented with a functional paraplegia of the left leg and high-grade paresis in the right leg associated with urinary retention. Sensory testing revealed impairment below Th5. Magnetic resonance imaging (MRI) of the thoracolumbar spine revealed multiple lesions of the vertebral bodies with an intraspinal mass lesion at level Th4/5 with subsequent spinal cord compression (Figs. 1, 2, 3 and 4).

The patient underwent emergency surgery for spinal decompression at levels Th4/5. Therefore, a dorsal approach with left-sided hemilaminectomy of Th4 and 5 with undercutting to the contralateral side and flavectomy was performed. The epidural tumor was solid and of brown-greyish appearance and could be removed completely. In the recessed part, tumor growth continued into the vertebral body. Here, diffuse bleeding could be controlled and the lateral aspect of the spinal cord and the evolving nerve roots were decompressed bilaterally. At the end of surgery, the spinal cord was pulsating and there was no compression of the myelon at the exposed levels. The postoperative course was uneventful. The neurological status of the patient improved and the paraparesis regressed but he still could not stand nor walk without assistance.

## Imaging

Computed tomography scans of the thoracic spine in bone window setting showed increased density of large portions of the vertebral bodies of Th4 and Th5 (Fig. 1, arrowheads). The vertebral body of Th5 showed a lytic area in its ventral portion and a compression fracture (Fig. 1, arrow). On sagittal T2-weighted images (Fig. 2) patchy heterogeneous signal intensities were observed in the vertebral body of Th5 (Fig. 2, arrow) as well as within various other vertebral bodies of the thoracic spine (Fig. 2, asterisks). An intraspinal, perimedullary lesion was found (Fig. 2, arrow) that showed an isointense signal when compared to the myelon. Corresponding findings were displayed on non-enhanced sagittal T1-weighted images (Fig. 3a). The vertebral body of Th5 (Fig. 3a, arrow) as well as various other vertebral bodies of the thoracic spine showed a marked decrease in signal intensities (Fig. 3a, asterisks). The intraspinal, perimedullary lesion clearly visible on T2-weighted images was hardly perceptible on sagittal native T1-weighted images (Fig. 3a, arrowhead). On non-enhanced axial T1-weighted images (Fig. 3b) at the level of Th5 the lesion appeared to be located in the epidural space (Fig. 3b, arrow) and showed infiltration of the vertebral body (Fig. 3b, arrowhead). After administration of gadolinium the affected vertebral bodies showed a marked yet patchy pattern of enhancement on sagittal T1-weighted images (Fig. 4a, arrow and asterisks). The epidural lesion exhibited homogeneous contrast enhancement and displaced the myelon ventrally (Fig. 4a, arrowhead). On axial T1-weighted postcontrast images the epidural portion of the lesion compressed the myelon (Fig. 4b, arrow), and the infiltration of the vertebral body (Fig. 4b, arrowhead) could be appreciated.

## Differential Diagnosis

### Spinal Metastasis

Spinal metastases (SM) are the most common malignant spinal lesions [1] and they are present in 10% of newly-diagnosed cancers. They are much more frequent in higher age groups (>50 years) without gender predilection. Clinical presentation is unspecific and ranges from asymptomatic to bone pain, pathological compression fractures, or extension into the spinal canal with cord compression and ensuing neurological deficits.

Spinal metastases most frequently derive from breast and lung cancer, prostate cancer and lymphoma [2]. Most SM arise at the lumbar or thoracic level, whereas a cervical location is less common. More than 50% of SM involve multiple levels. The MRI findings of SM vary greatly. A solitary focal round/ovoid mass with strong contrast enhancement and multiple lesions is a common presentation. In T1-weighted images SM typically appear as focal or diffuse hypointense vertebral body lesions. The disc space is usually preserved. In T2-weighted images most metastases appear isointense to hyperintense and sclerotic metastases appear hypointense, which is in line with the diagnostic findings of our patient. In patients over 50 years of age, metastases always have to be considered as a potential differential diagnosis [3].

### Multiple Myeloma (MM)

Multiple myelomas are multifocal malignant proliferations of monoclonal plasma cells within the bone marrow with a peak incidence between mid-60 years old to early 70 years old and a male predilection of 2:1 [4]. With an incidence of 6 per 100,000 people per year, MM is a common bone tumor in adults and accounts for 27% of biopsied bone tumors [4, 5]. Clinical presentation is variable and includes bone pain, pathologic fractures and symptoms related to bone marrow failure, such as anemia, infections or weight loss, whereas 20% remain asymptomatic [4]. Typical laboratory abnormalities including: reverse albumin/globulin ratio, monoclonal Bence Jones protein, proteinuria, hypercalcemia, decreased or normal alkaline phosphatase (ALP) unless there is a pathological fracture due to impaired osteoblastic function [4].

On MRI imaging these tumors present with normal, focal, diffuse or variegated (salt and pepper) marrow involvement. Magnetic resonance imaging typically shows on T1-weighted images focal or diffuse hypointensities of vertebral body or a variegated pattern with pathologic compression fractures, predominantly between Th6 and L4 [6]. The imaging features of the vertebral bodies in our patient could be in line with the diagnosis of multiple myeloma, yet in the absence of defining laboratory abnormalities, the diagnosis of MM seemed less likely.

### Chronic Recurrent Multifocal Osteomyelitis

Chronic recurrent multifocal osteomyelitis (CRMO) is an autoinflammatory disorder of the bones with fever, multifocal painful non-pyogenic bone lesions, relapsing and remitting course in children and adolescents. The CRMO is part of a heterogeneous group of disorders of the innate immune system with a female predominance and a great variety of age ranging from infants up to 55 years of age with a median age of 9 years. Only 15% of lesions are accompanied by a spinal involvement [7]. Back pain, scoliosis, kyphosis and rarely symptoms of cord compression due to multiple bone lesions are the most common symptoms.

In T1-weighted images focal lesions with low signal intensities and loss of vertebral body height are found as well as normal signals of the intervertebral discs. The T2-weighted images reveal focal lesions of the vertebral bodies with increased signal intensity, whereas the intervertebral discs again are spared [8]. Disregarding the advanced age of our patient, he met with imaging-proven osteolytic/sclerotic bone lesion and multiple bone lesions some major diagnostic criteria of CRMO. Palmoplantar pustulosis or psoriasis and a sterile bone biopsy were not present.

### Tuberculous Osteomyelitis

Tuberculosis (TB) osteomyelitis is a rare musculoskeletal manifestation of TB and accounts for 2% of all TB cases with a trend towards a rising incidence in the past two decades [9]. The TB osteomyelitis is most prevalent in the 5th decade of life without gender predilection. Patients may present with chronic back pain, focal tenderness, and fever but also paraparesis, kyphosis, sensory disturbance, bladder and bowel dysfunction [10].

Imaging may show a gibbus deformity of the vertebrae with relatively intact intervertebral discs and often large paraspinal abscesses over a considerable distance. The predominant location of the lesions is midthoracic or thoracolumbar. On MRI TB osteomyelitis presents with hypointense marrow signal in contiguous vertebrae, hypointense intraosseous, extradural and paraspinal abscesses on T1-weighted images, and with hyperintense marrow, disc, phlegmon/abscess in T2-weighted images [11]. The lack of typical gradual onset of symptoms of TB osteomyelitis, and the absence of paraspinal abscesses make this diagnosis less likely.

### Spinal Lymphoma

Lymphomas are malignancies that develop from lymphocytes and are typically located in lymph nodes. Primary bone lymphoma (PBL) is an uncommon extranodal manifestation of a lymphoma that represents about 1–3% of lymphomas. Cases of PBL located in the spine are even rarer and PBL has a male predilection. The peak incidence is the 5th–7th decades of life [12]. The majority of PBL of the spine are located at the thoracic level followed by the lumbar and the cervical spine. Chronic back pain is the most common presenting symptom, with neurological signs appearing later in the disease course. In a minority of patients B symptoms can also be present [13].

Computed tomography (CT) imaging shows an epidural homogeneous, slightly hyperdense mass, with or without bone involvement. In the bone window there is lytic permeative bone destruction and multilevel involvement which crosses disc spaces. On T1-weighted images isointense, homogeneous epidural masses (often multisegmental ± extend through foramina) can be found. In addition, the affected vertebral bodies display a hypointense signal compared to normal marrow (± epidural extension). On contrast-enhanced images, lesions show a marked enhancement. On T2-weighted images we find an epidural lesion which appear isointense/hyperintense to the spinal cord. The vertebral bodies show variable isointense or hyperintense signals [14].

This entity constitutes a diagnostic challenge because it may mimic other spinal diseases, such as spinal and vertebral metastases. In addition, it can prove difficult to establish a tissue diagnosis in these patients. In fact, core biopsy can be inconclusive and often requires surgical biopsy.

## Histology

### Histology and Immunohistochemistry

**Fig. 5:**
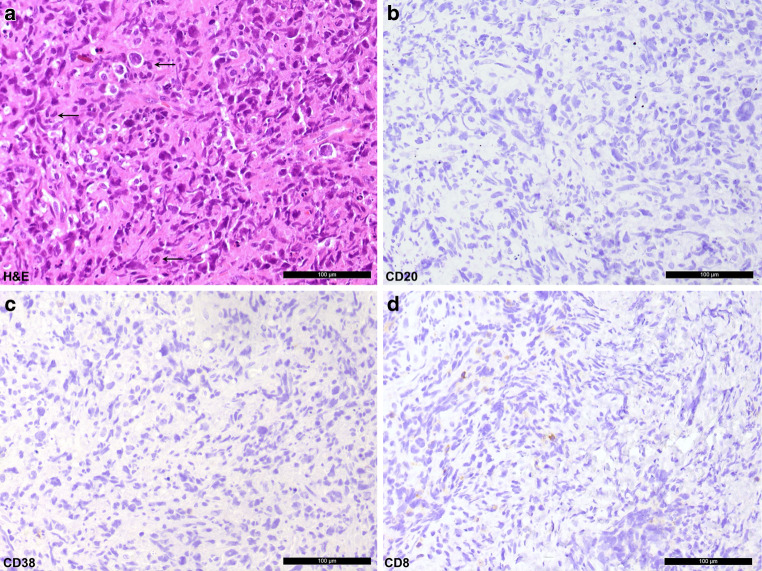
Hematoxylin and eosin stained section (**a**) showing a hematopoietic tumor with high cellularity. Eccentric, kidney-shaped nuclei are marked with arrows. Immunohistochemistry for CD20 indicative for B cells (**b**), CD38 for plasma cells (**c**), and CD8 demonstrating cytotoxic T cells (**d**). Size bar 100 µm

A biopsy of an intraspinal epidural tumorous growth was obtained. The patient had no history of cancer prior to the current biopsy. The hematoxylin-eosin (H&E) stained sections of the formaldehyde-fixed and paraffin-embedded biopsy specimen presented a hematopoietic tumor with high cellularity exhibiting predominantly large pleomorphic cells of lymphocytic morphology with interspersed nuclei (Fig. 5a). Some eccentric kidney-shaped nuclei were seen (Fig. 5a). The tumor cells exhibited a cohesive growth pattern. Mitotic figures and apoptotic cells were observable and necrosis was present focally.

**Fig. 6:**
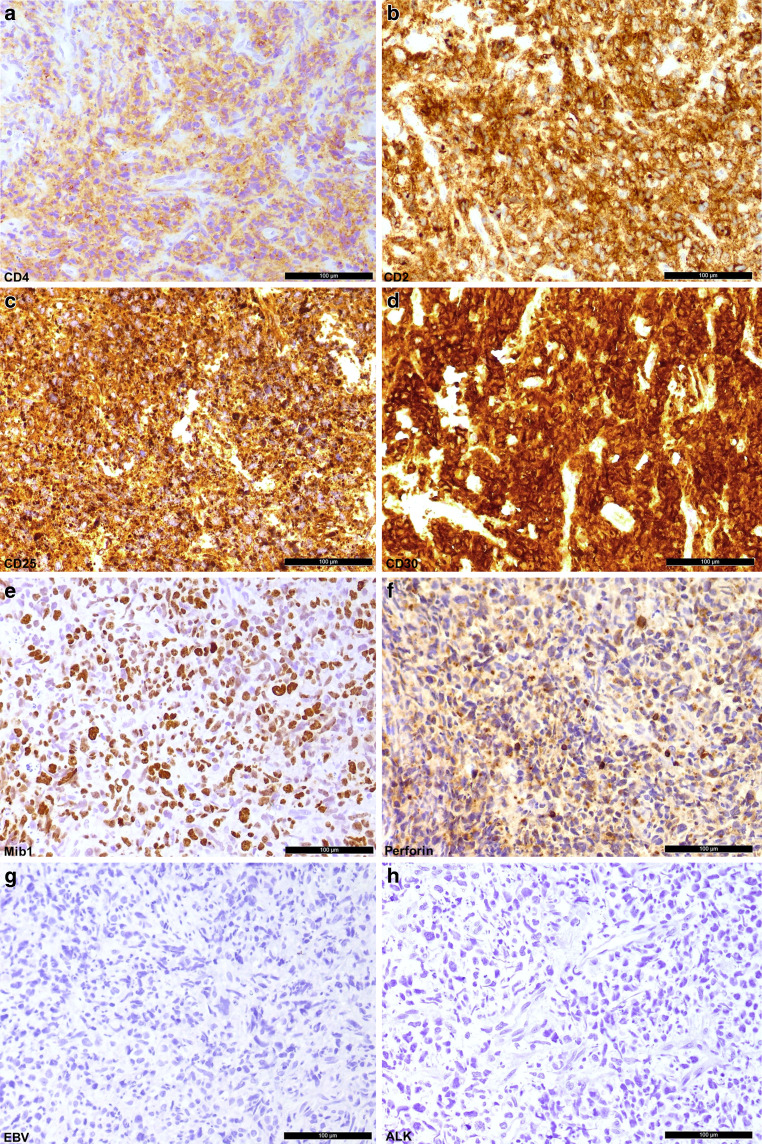
Tumor cells show positive labeling for CD4 (**a**), CD2 (**b**), CD25 (**c**) and CD30 (**d**). The proliferation index is increased tagging more than 80% of tumor cells, depicted by the MIB1 staining (**e**). Perforin is expressed by the tumor cells (**f**). Epstein-Barr virus (EBV) was not observable in the tumor cells (**g**). Anaplastic lymphoma kinase (ALK) expression remained negative (**h**). Size bar: 100 µm

Immunohistochemistry for CD20 indicative for B cells, CD38 marking plasma cells, and CD8 demonstrating cytotoxic T cells did not stain tumor cells positively (Fig. 5b–d). The immunohistochemical reactions against CD4, CD2, CD25 and CD30 stained the majority of cells positively (Fig. 6a–d). Staining for proliferative capacity with Mib1 (Ki67) revealed up to 80% of cells as proliferative (Fig. 6e). Perforin was expressed by the tumor cells (Fig. 6f), while other cytotoxicity-associated markers (granzyme B and TIA1 [Tia1 cytotoxic granule-associated rna binding protein]) remained negative (data not shown). As expected, the tumor cells were negative for Epstein-Barr virus (EBV) (Fig. 6g). Anaplastic lymphoma kinase (ALK) expression was not identifiable (Fig. 6h).

The findings suggest a diagnosis of a T cell lymphoma consistent with an anaplastic large cell lymphoma. Differential diagnoses comprise an anaplastic lymphoma kinase-(ALK)-positive anaplastic large cell lymphoma; however, immunophenotypic negativity for ALK argues against this entity. Furthermore, a peripheral T cell lymphoma not otherwise specified (NOS) should be taken into account; however, according to the World Health Organization (WHO) recommendations both the morphology and phenotype of the current biopsy support the diagnosis of an ALK-negative, anaplastic large cell lymphoma [15]. The diagnosis is further supported by detection of the *IRF4/DUSP22* translocation [16] and was confirmed independently by the institute of neuropathology Cologne, Germany.

## Diagnosis

### Anaplastic Large Cell Lymphoma of the Spine

The most common form of lymphoma affecting bones is the diffuse large B cell lymphoma (DLBCL), which accounts for approximately 5% of all extranodal lymphomas and 2–3% of all CNS tumors [15, 17–19]. The entity described in the current case has an intraspinal and extramedullary location affecting the vertebral body and presented features of a T cell neoplasm. In general, anaplastic large cell lymphoma is a distinct entity of non-Hodgkin lymphoma [15]. Anaplastic large cell lymphoma are typically located in lymph nodes and extranodal manifestations are less common; however, isolated bony or spinal localizations as in the current case have been reported [17, 20–23]. Patients suffering from diffuse large cell lymphoma, ALK-positive are reported to have a favorable prognosis compared with diffuse large cell lymphoma, ALK-negative [15, 24].
